# Aquatic top predator prefers terrestrial prey in an intermittent stream

**DOI:** 10.1002/ecy.4518

**Published:** 2025-01-21

**Authors:** Amin M. al‐Jamal, Albert Ruhi, Rose M. Mohammadi, Michael T. Bogan, Robert J. Fournier

**Affiliations:** ^1^ Department of Environmental Science, Policy, and Management University of California, Berkeley Berkeley California USA; ^2^ Institute for Biodiversity Science and Sustainability California Academy of Sciences San Francisco California USA; ^3^ School of Natural Resources and the Environment University of Arizona Tucson Arizona USA

**Keywords:** foraging, giant water bug, intermittent stream, perimeter to area (P/A) ratios, predation

Trophic interactions often span traditional habitat boundaries or “edges” (Strayer et al., 2003). This is particularly true in ecosystems with high perimeter to area (P/A) ratios such as oceanic small islands, which receive strong allochthonous resource flows from marine‐derived nutrients, detritus, and organisms relative to their own autochthonous (local plant matter) production (Polis & Hurd, 1996). Small waterbodies surrounded by terrestrial habitat share similar traits, with aquatic predators often seasonally relying on allochthonous terrestrial prey (Nakano & Murakami, 2001). Intermittent streams and rivers that experience seasonal cycles of drying are highly prevalent across the globe (Messager et al., 2021), but important questions around their food‐web dynamics remain (McIntosh et al., 2017). These systems often feature large arthropods, not fish or amphibians, as the top aquatic predators, and hydrologic variation largely controls their food‐web structure (Ruhí et al., 2017; Sabo et al., 2010). Here, we investigated the propensity of a giant water bug, *Abedus*, to prey preferentially on terrestrial taxa in fishless, intermittent streams and discuss the significance of this preference in the context of ecosystems that have highly fluctuating P/A ratios.

Giant water bugs (Hemiptera: Belostomatidae) are predatory insects with a widespread distribution among fresh waters. Most species prefer slow‐moving or lentic habitats and are often an abundant top predator (Swart & Taylor, 2004). Giant water bugs capture prey using their sharp raptorial forelegs and feed by piercing the prey's body with a thick jointed stylet that injects a mixture of digestive and paralytic enzymes (Ohba, 2019; Figure 1A). This grappling‐piercing mechanism allows them to catch and consume relatively large prey, resulting in a high predation success rate (Figure 1B,C). While they are assumed to feed opportunistically, some studies have suggested that they may prefer less agile, defenseless prey (Velasco & Millan, 1998).

**FIGURE 1:**
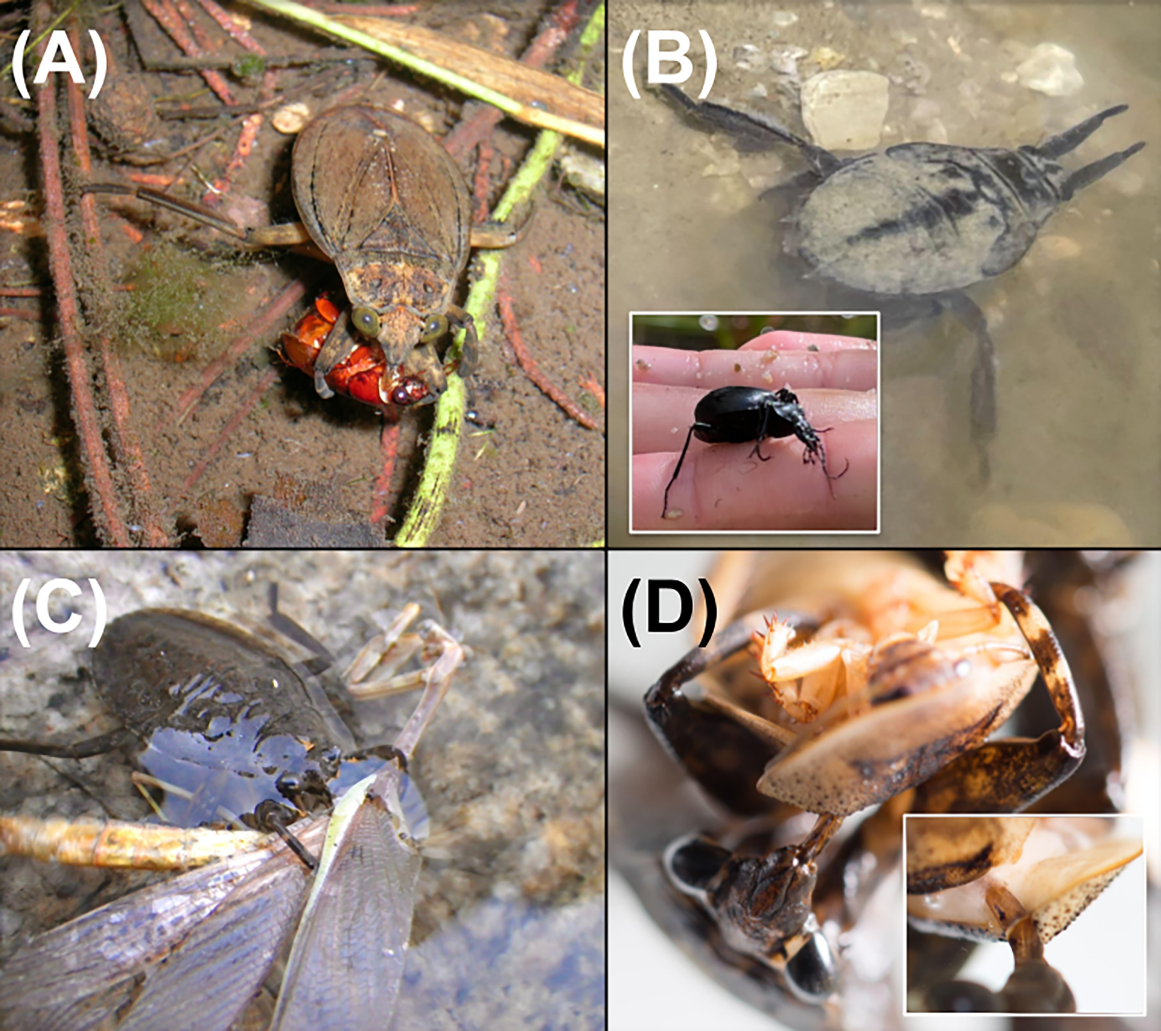
Field observations of *Abedus*' trophic interactions. (A) *Abedus herberti* consuming a terrestrial beetle (cf. *Phyllophaga* sp.) in Cave Creek, Arizona; (B) *Abedus indentatus* found on a margin of an isolated pool at Chalone Creek, Pinnacles National Park, California, in lie‐and‐wait position. Inset: Scaphonotus carabid beetle that *Abedus* captured and later released at the same site when we handled *Abedus* during aquatic invertebrate sampling; (C) *A. herberti* consuming a mantid (cf. *Stagmomantis limbata*) in Santa Catalina Mountains, Arizona; (D) *A. indentatus* feeding on restrained *Blaptica dubia*. Inset shows stylet piercing between sclerites. Photo credits: (A, C): Michael T. Bogan; (B): Albert Ruhi; (D): Amin M. al‐Jamal.

Our observations arise from more than 20 field trips over the last decade to Pinnacles National Park (central California, USA), the ancestral homelands of the Amah Mutsun and Chalone peoples. The Park is characterized by a semiarid Mediterranean climate and a stream network that dries seasonally across approximately 98% of its length. This highly fluctuating hydrology has promoted drought‐resistant and resilient animal communities (Fournier et al., 2023). The belostomatid giant water bug *Abedus indentatus* is the dominant predator in fishless sections of the river network (i.e., the intermittent and ephemeral reaches), similar to the top predator role that its congener *Abedus herberti* plays in US Southwest desert streams (Smith, 1974).

In the field, we repeatedly observed *A. indentatus* feeding on a variety of terrestrial prey, including beetles, orthopterans (Gryllidae, Tettigoniidae), Mantodea, and various flying Hymenoptera (Figure 1A). We also observed *A. indentatus* feed on large aquatic prey, such as land‐locked anurans and three‐spined stickleback (*Gasterosteus aculeatus*). Notably, these observations invariably occurred during the drying phase (in late spring), when isolated pools are compressed and the stream P/A ratio reaches its peak. In those conditions, *Abedus* wait in shallow waters for a chance to attack flying insects that land on the surface (Figure 1B). The observed frequency of *Abedus* feeding on terrestrial organisms was high, suggesting that they may prefer terrestrial organisms when available. We sought to test this hypothesis experimentally, by presenting aquatic and terrestrial prey across a range of sizes to *A. indentatus* under controlled conditions in a laboratory setting. In addition, we examined δ^13^C isotopic signatures from wild‐caught individuals (*N* = 12) of two aquatic predators (Belostomatidae and Aeshnidae) and one algivorous grazer (Lymnaeidae) to assess whether preference in laboratory conditions reflected predatory patterns in natural systems. In this case, depleted δ^13^C values (i.e., more negative) would indicate reliance on autochthonous algal energy pathways, while higher (i.e., less negative) δ^13^C values reflect reliance on terrestrially derived carbon sources.

To detect patterns in prey preference with respect to size and origin (aquatic or terrestrial), we used a “prey‐drop” assay similar to that of Velasco and Millan (1998). This design facilitates inference of prey preference when a predator is simultaneously presented with a suite of prey—each representing a different combination of nutritional values and defensive abilities. We gathered 44 specimens of *A. indentatus* from Chalone Creek in Pinnacles National Park, California, in summer 2022. We selected prey items that represented a range of sizes as well as gradients of agility and physiological defense. Roughly half of the potential prey choices are primarily aquatic, while the remainder are terrestrial (see Appendix S1: Table S1 for taxa list). All prey types were naturally present in the system except for cockroaches (Blaptica), which have been previously used as a model for terrestrial, palatable prey in feeding studies (e.g., Veselý & Fuchs, 2009).

Each individual *A. indentatus* was tested in a 30 × 20 × 20‐cm acrylic aquarium fitted with a diagonal wooden perch in a laboratory setting. Food was withheld for 48 h prior to testing to ensure common initial conditions. Similar diet experiments on other belostomatids have used starvation periods of 2–7 days (see, e.g., Ohba & Takagi, 2005). At the beginning of each assay, one *A. indentatus* was placed into the test arena alone for 10–15 min. Following this acclimation period, a full set of live prey items (one of each type) was dropped simultaneously into the observational arena (Appendix S1: Figure S1). We ran a total of 31 trials (one per predator), with three assays running concurrently at any one time. Each trial ran for 2 h during daylight. Captured prey items were taken away from predators after 5 min, using forceps. This prevented *A. indentatus* from feeding on a single prey item for the entire observational period, thus revealing subsequent prey preference. For each assay, we quantified the binary response (attack or no attack) to each prey item, and the order in which prey were captured.

We used Plackett–Luce models to infer the worth or profitability (sensu MacArthur & Pianka, 1966) of the different prey to *A. indentatus*. These models compare all prey items to each other, accounting for the sequential removal of previously selected items (Turner et al., 2020). Thus, the *worth* metric represents the preference of each predator for each prey item. To test whether prey size influenced selection probability, we calculated prey mean length by measuring 10 individuals of each prey species used in the trials. From these means, we used length–mass relationships to calculate prey dry mass (Appendix S1: Table S3). We fitted logistic regressions for the binary attack outcome (attacked or not) against prey mean length and mass. Statistical analyses were done in R using an ɑ of 0.05.

We found that terrestrial prey were selected more often than aquatic prey: 69.4% of the terrestrial prey, but only 41.9% of the aquatic prey, were selected across trials (χ^2^ = 24.812, df = 1, *p* < 0.001; Figure 2A). Additionally, large organisms from both terrestrial and aquatic categories constituted a high proportion of first strikes (Figure 2A) as strike likelihood increased with prey size (length: *estimate* = 0.0616, df = 340, *p* = 0.001; mass: *estimate* = 0.0326, df = 340, *p* < 0.001). The Plackett–Luce models confirmed that large terrestrial prey had the highest estimated worth, and small aquatic prey items had the lowest estimated worth (pairwise *Z*_359_ = 2.002, *p* = 0.045; Figure 2B). In short, larger prey are more likely to be attacked. However, patterns differed between aquatic and terrestrial prey. The attack likelihood of aquatic prey increased with prey size (length: *estimate* = 0.12797, df = 154, *p* < 0.001; mass: *estimate* = 0.0323, df = 154, *p* = 0.0028). Conversely, no such relationship was found for terrestrial prey (length: *estimate* = 0.0112, df = 184, *p* = 0.626; mass: *estimate* = 0.0152, df = 185, *p* = 0.273; Figure 2C). Importantly, isotopic signatures confirmed that Belostomatid predators in the wild have less depleted δ^13^C signals (−27.5 ± 1.39) than predators that are known to rely on aquatic prey, such as Aeshnidae odonates (−31.4 ± 0.608). Notably, δ^13^C values of Aeshnidae, but not of *A. indentatus*, were similar in range to those of an abundant periphyton grazer in the system, Lymnaeidae snails (−30.6 ± 1.525; Figure 2D; Appendix S1: Figure S3).

**FIGURE 2:**
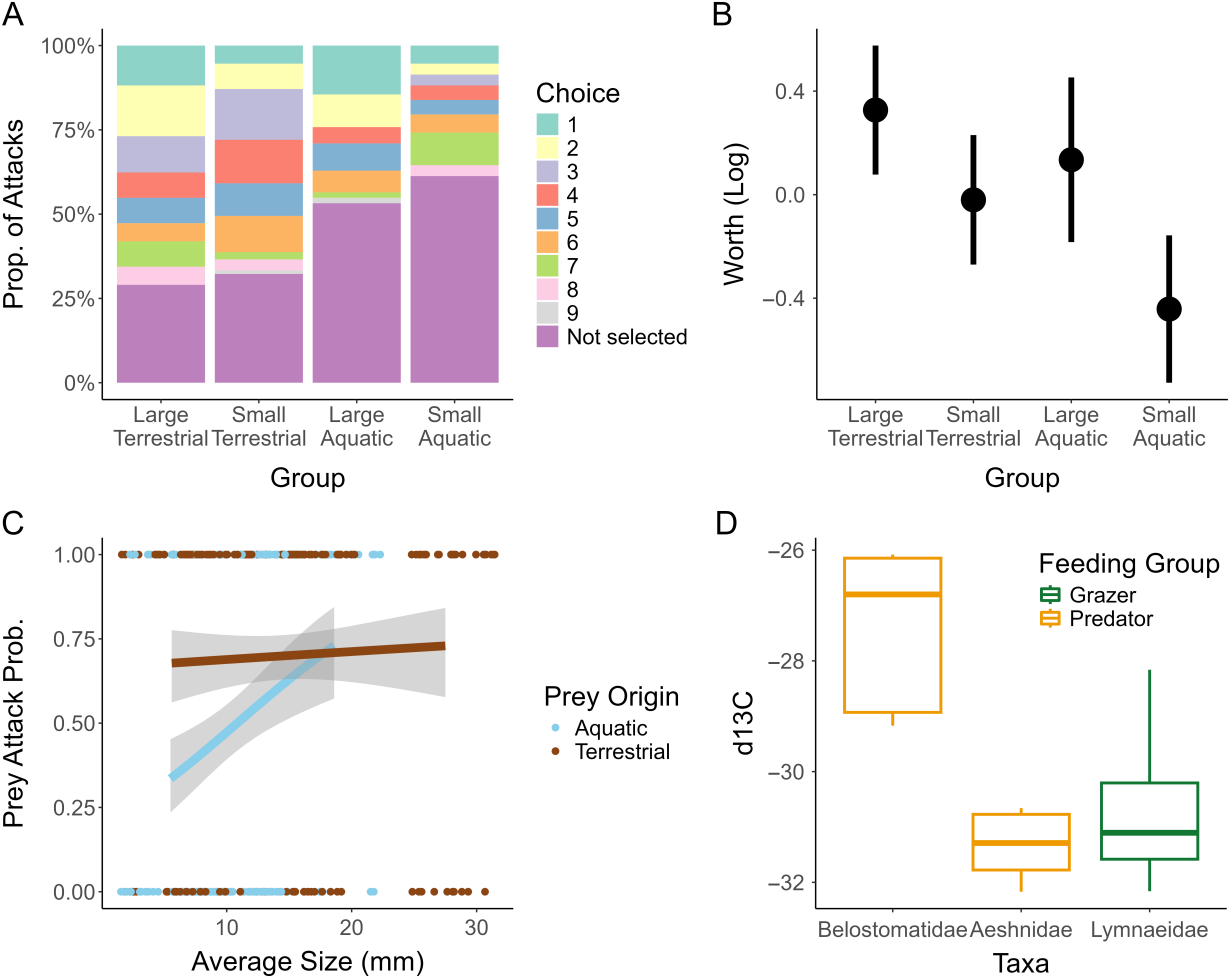
Results on *Abedus*' prey preference and reliance. (A) Proportion of predation events by prey group. Choice indicates the order in which a given prey item was attacked. (B) Plackett–Luce estimation of worth (on the log scale) for each prey group. Error bars represent quasi‐SEs. Quasi‐SEs that overlap with each other indicate statistically similar estimates of worth. (C) Logistic regressions for mean size of aquatic and terrestrial prey items (*n* = 341) against whether a given prey item was attacked during an assay (0 for not attacked, 1 for attacked). Points are jittered (*w* = 0.6) along the *X*‐axis to accurately represent sample size. (D) δ^13^C Isotopic signatures for wild‐caught taxa at Pinnacles National Park (4 individuals each, 3 analytical replicates, total *N* = 36). Higher (i.e., less negative) δ^13^C values indicate a stronger influence of terrestrially derived carbon.

Collectively, these results support our hypothesis that when both aquatic and terrestrial prey are available, *A. indentatus* prefer the latter. Terrestrial insects that fall into aquatic habitats often lack adaptations to survive on the water surface or underwater and fail to adopt defensive behaviors to an unrecognized aquatic predator—becoming highly vulnerable to predation (Sih et al., 2010). Thus, a lack of morphological or behavioral adaptations to resist an aquatic predator might increase the profitability of terrestrial prey by decreasing predators' searching and handling times (Mohd & Noorani, 2021). We also observed that predation risk increased with prey size for aquatic prey, but not for terrestrial prey (Figure 2C), suggesting that even if *A. indentatus* favor larger prey (Venkatesan & D'sylva, 1990), prey origin might supersede this preference (Klecka & Boukal, 2012).

While freshwater fish commonly rely on terrestrial prey, and sometimes even seasonally prefer them (Nakano et al., 1999), such foraging patterns remain largely unknown from invertebrate predators. Other semiaquatic Hemiptera such as water striders (Gerridae) and backswimmers (Notonectidae) might opportunistically target drowning terrestrial prey. However, a strong reliance (or even preference) for terrestrial prey is rare in aquatic insects (e.g., Tachet et al., 2010; Vieira et al., 2006; but see Carlson et al., 2020). We contend that this mismatch may at least partly reflect the fact that drying‐prone stream ecosystems, and the adaptations of the invertebrate communities they host, have been understudied compared to their temperate counterparts (Bonada & Dolédec, 2011; Datry et al., 2014).

In ecosystems with high P/A ratios such as small streams, allochthonous inputs such as detrital leaf litter and drowning terrestrial insects can strongly support aquatic consumers (as previously described in Baxter et al., 2004; Nakano et al., 1999). In our case, intermittent streams during the dry phase are longitudinally fragmented, and isolated pools reach very high P/A ratios. For instance, P/A ratios of intermittent pools at Pinnacles ranged from ~1 to 4 m/m^2^ (or ~1000–4000 in units of kilometers per square kilometer, following the convention of Polis et al., 1998). These values are approximately 5–20 times larger than those typically observed in vernal pools (Brooks & Hayashi, 2002), 6–100 times larger than those observed in oceanic islands (Polis et al., 1998), and 100–350 times larger than those observed in lakes globally (Messager et al., 2016) (Appendix S1: Figure S4A). Notably, P/A ratios changed substantially within intermittent stream pools over the drying season, increasing on average by 73%. Thus, intermittent stream pools are not only substantially smaller but also more highly connected on average than most ecosystem types—particularly during the dry season (Appendix S1: Figure S4B). Consequently, we contend that an increased availability of terrestrial prey facilitated by a high proportion of edge habitat, coupled with the increased susceptibility of terrestrial prey crossing the terrestrial–aquatic boundary, allowed *A. indentatus* to develop an uncommon dietary preference among stream insects.

The results of our field observations and feeding experiment suggest at least two lines for future research. First, while we showed that *A. indentatus* prefers terrestrial prey, effects on fitness remain unknown. Prey quality extends beyond net caloric gain, and variation in the type, concentration, or total mass of key nutrients between terrestrial and aquatic prey could affect *A. indentatus*' performance. Studies that focus on the effects of these subsidies—for *A. indentatus* and the broader food web—are warranted. Second, P/A ratios in intermittent river networks peak in the drying phase, when pools become isolated and wet habitat contracts. Tracking changes in aquatic predator reliance on terrestrial prey over time (during the dry, rewetting, and wet hydrologic phases) would allow testing to what extent variation in P/A ratios drives allochthony and facilitates comparisons to well‐established literature on the “fixed” relationship between island size and subsidy‐driven productivity (Polis & Hurd, 1996). Overall, our results add to growing evidence that aquatic–terrestrial linkages may be particularly important in ecosystems with abundant edge habitat such as intermittent freshwaters and illustrate the need for conservation strategies that account for biotic interactions across the aquatic‐terrestrial interface.

## AUTHOR CONTRIBUTIONS

Amin M. al‐Jamal, Albert Ruhi, Michael T. Bogan, and Robert J. Fournier provided field observations that form the basis of the study. Amin M. al‐Jamal and Albert Ruhi designed the diet experiment. Amin M. al‐Jamal acquired the data. Rose M. Mohammadi acquired the isotopic data. Robert J. Fournier and Rose M. Mohammadi performed the data analysis. Robert J. Fournier and Amin M. al‐Jamal drafted the manuscript. Albert Ruhi and Robert J. Fournier revised the manuscript. All authors approved the manuscript.

## CONFLICT OF INTEREST STATEMENT

The authors declare no conflicts of interest.

## Supporting information


Appendix S1.

## Data Availability

Data (al‐Jamal et al., 2024a) are available in Dryad at 10.6078/D1FB14. Code (al‐Jamal et al., 2024b) is available in Zenodo at 10.5281/zenodo.8190455.
