# Supplementary material for: Aquatic top predator prefers terrestrial prey in an intermittent stream
Source: Ecology. 2025 Jan 21;106(1):e4518. doi: 10.1002/ecy.4518 (PMC11750761; doi:10.1002/ecy.4518)
Supplement: Supplementary file 1 — Appendix S1. [file ECY-106-e4518-s001.pdf]

Ecology

Aquatic top predator prefers terrestrial prey in an intermittent stream

Amin M. al-Jamal, Albert Ruhi, Rose M. Mohammadi, Michael T. Bogan, Robert J. Fournier

Supporting Information - Appendix S1

Section S1: Supplementary methods:

Additional information on specimen collection

To capture our specimens, we conducted serial edge sampling and pool sweeps (3 each) using a 5 mm mesh net at different sites of Chalone Creek, Pinnacles National Park, California (Lat: 36.46584, Long: -121.15532). Specifically, we gathered forty-four specimens of *Abedus indentatus* on May 25 and June 21, 2022. Predators were kept individually and transported to the lab in large collection jars with mesh tops, containing stream water and perches. Predators were fed *Tenebrio molitor* larvae prior to experiments but starved for 48 hours prior to trials.

In turn, aquatic prey were collected on open space in San Geronimo, California, United States (Lat: 38.01363, Long: -122.65289) using serial edge sampling and pool sweeps, Terrestrial prey were mostly captured via aerial netting, sweep-netting tall grass, and beating coast live oak, except for *Acheta domestica* and *Blaptica dubia*, which were purchased (see Table S1 for complete prey list and origins).

Finally, taxa representing food-web bookends were collected for stable isotope analysis ($\delta^{15}N$, $\delta^{13}C$) along the same creek branch where the *Abedus* specimens were collected. We focused on Lymnaeidae snails (Gastropoda), an epilithic grazer that should capture the 'green'

energy pathway, and Aeschnidae dragonflies (Odonata), the top predator alternative to *Abedus* in fishless, intermittent stream sections. We hypothesized this predator would also assimilate the 'green' energy pathway, given their strong reliance on grazer insect larvae. Finally, we collected additional specimens of the target belostomatids. We collected four specimens of each taxa, which were transported to the lab in cold conditions (~10 degrees C) for 24 hours, to evacuate gut contents. Specimens were posteriorly dried at 55 degrees C, ground, and transferred to tin capsules in sets of analytical triplicates. $\delta^{15}N$, $\delta^{13}C$ analyses were ran at the Center for Stable Isotope Biogeochemistry on the UC Berkeley campus.

Statistical analyses

Placket-Luce models use Luce's axiom (Luce 1977), where the probability of selecting any *j* item from a given set (*S*) is:

$$P(j|S) = \frac{\alpha_j}{\Sigma_{i\epsilon S}\alpha_i}$$

where α represents the worth of item *i*. As prey items were removed once selected, the sequential probabilities (and estimated *worth*) of choosing an item once the previously selected item has been removed, follow the equation:

$$\prod_{j=1}^{j} \frac{\alpha_{ij}}{\sum_{i\varepsilon A_i} \alpha_i}$$

where A_j is the set of alternatives from which item i_j is chosen. We used the PlackettLuce R package (Turner et al. 2020) to calculate estimates of worth for each prey item and associated quasi standard errors.

Section S2: Supplementary Tables

Table S1: Information regarding prey items in our feeding experiment.

**Scientific name**	**Common name**	**Source**	**Mean size (mm ± SD)**	**Size category**	**Origin**
Acheta domestica	Cricket (large)	Purchased	16.7 ± 1.41	Large	Terrestrial
Blaptica dubia	Cockroach	Purchased	15.1 ± 1.7	Large	Terrestrial
Ischnura sp. (adult)	Adult damselfly	Wild caught	27.5 ± 2.10	Large	Terrestrial
Acheta domestica	Cricket (small)	Purchased	8.2 ± 1.16	Small	Terrestrial
Diabrotica undecimpunctata	Cucumber beetle	Wild caught	5.6 ± 0.91	Small	Terrestrial
Forficula auricularia	Earwig	Wild caught	12.2 ± 1.66	Small	Terrestrial
Belostoma flumineum	Giant water bug	Wild caught	18.5 ± 2.06	Large	Aquatic
Notonecta sp.	Backswimmer	Wild caught	11.4 ± 1.2	Large	Aquatic
Dytiscidae sp.	Diving beetle	Wild caught	5.5 ± 0.5	Small	Aquatic
Corixidae sp.	Water boatman	Wild caught	6.8 ± 1.33	Small	Aquatic
Ischnura sp. (larvae)	Larval damselfly	Wild caught	10.7 ± 1.73	Small	Aquatic

Table S2: Approximate calorimetry values for prey taxa, based on values from the literature. If values were not species-specific, the reference taxa is listed.

**Prey taxa**	**Reference taxa**	**Origin**	**Calorimetric value**	**Source**
Forficula	Dermaptera	Terrestrial	25.94 J/mg	Ghenam and Bachir 2011
Dytiscidae	–	Aquatic	25.10 J/mg	Anderson and Smith 1998
Diabrotica	Chrysomelidae	Terrestrial	23.15 J/mg	Edwards and Wightman 1984
Belostoma	Belostomatidae	Aquatic	22.18 J/mg	Anderson and Smith 1998
Corixidae	–	Aquatic	22.16 J/mg	Clark 1992
Acheta	Gryllidae	Terrestrial	21.76 J/mg	Robel et al.1995
Blaptica	–	Terrestrial	21.64 J/mg	Hopley 2016
Ischnura	Coenagrionidae	Both*	20.08 J/mg**	Anderson and Smith 1998
Notonecta	Notonectidae	Aquatic	17.15 J/mg	Anderson and Smith 1998

*Ischnura are aquatic in their nymphal stages, and terrestrial in their adult stages.
**Calorimetric data represent nymphal stages.

Table S3: Length-Mass relationship coefficients used to estimate prey average biomass based on average length, and associated references. Here, *a* and *b* are constants in the length-mass equation with *a* representing the Y intercept and *b* representing the slope.

**Prey taxa**	**Origin**	***a***	***b***	**Source**
Forficula	Terrestrial	0.0015	3.497	Hodar et al. 1996
Dytiscidae	Aquatic	0.0618	2.502	Benke et al. 1999
Diabrotica	Terrestrial	0.0392	3.111	Benke et al. 1999
Belostoma	Aquatic	0.0376	2.417	Hodar et al. 1996
Corixidae	Aquatic	0.0031	2.904	Benke et al. 1999
Acheta	Terrestrial	0.03	2.55	Sabo et al. 2002
Blaptica	Terrestrial	0.0494	2.344	Hodar et al. 1996
Ischnura	Both*	0.0015	2.904	Benke et al. 1999
Notonecta	Aquatic	0.0376	2.417	Hodar et al. 1996

*Ischnura are aquatic in their nymphal stages, and terrestrial in their adult stages.

Section S3: Supplementary Figures

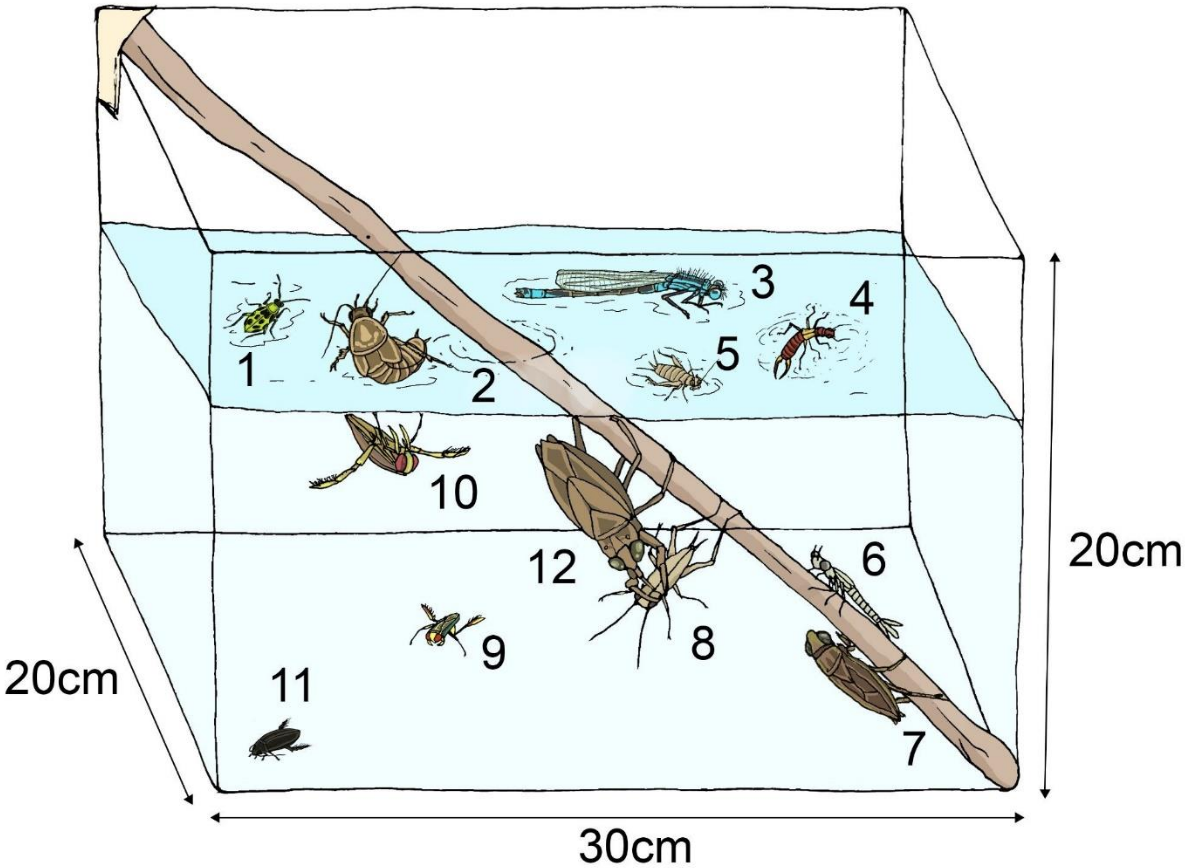

Figure S1: Diagram of the observational arena during a typical trial of the experiment. Terrestrial taxa often floated in the water while aquatic taxa exhibited a range of swimming behaviors. Taxa: 1, *D. undecimpunctata*; 2, *B. dubia*; 3, *Ischnura sp*. adult; 4, *F. auricularia*; 5, small *A. domestica*; 6, *Ischnura sp*. nymph; 7, A belostomatid nymph, *B. flumineum*; 8, large *A. domestica*; 9, Corixidae *sp*.; 10, *Notonecta sp*.; 11, Dytiscidae sp.; 12, *A. indentatus* (predator). Illustration credit: Amin M. al-Jamal.

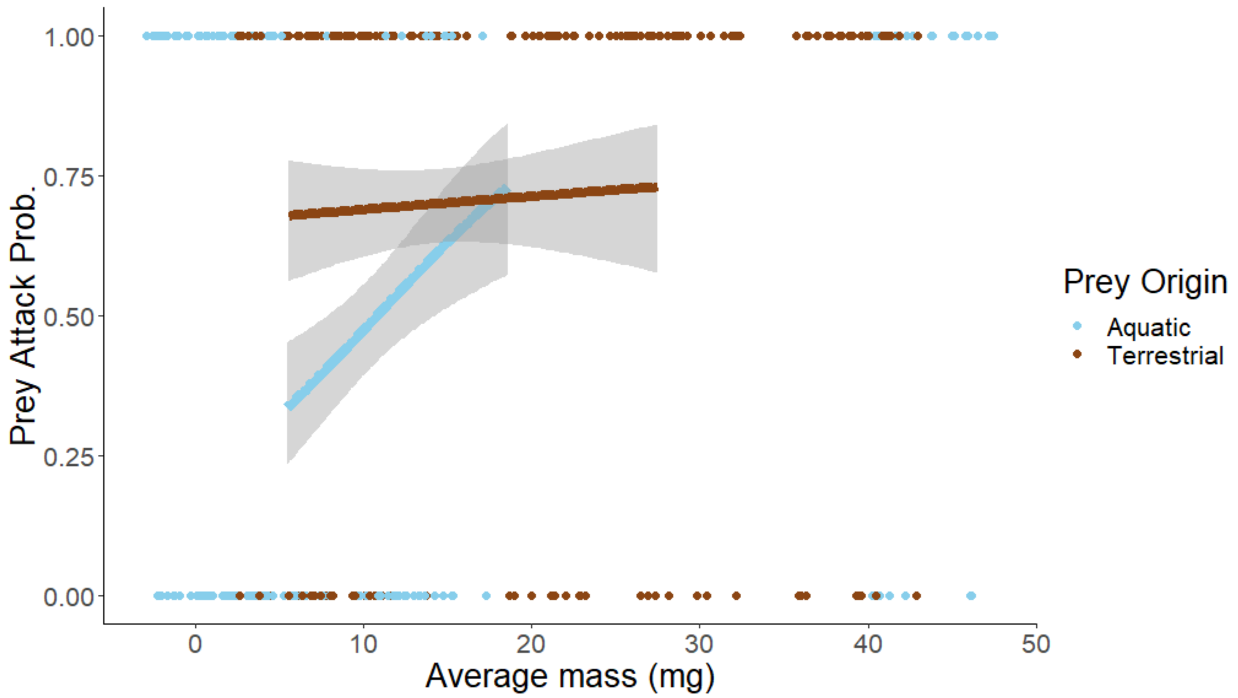

Figure S2: Logistic regressions for mean body mass of aquatic prey items and mean body mass of terrestrial prey items, against whether a given prey item was attacked or not during an assay (0 for not attacked, 1 for attacked). Points are jittered (w=0.6) along the X-axis to accurately represent sample size.

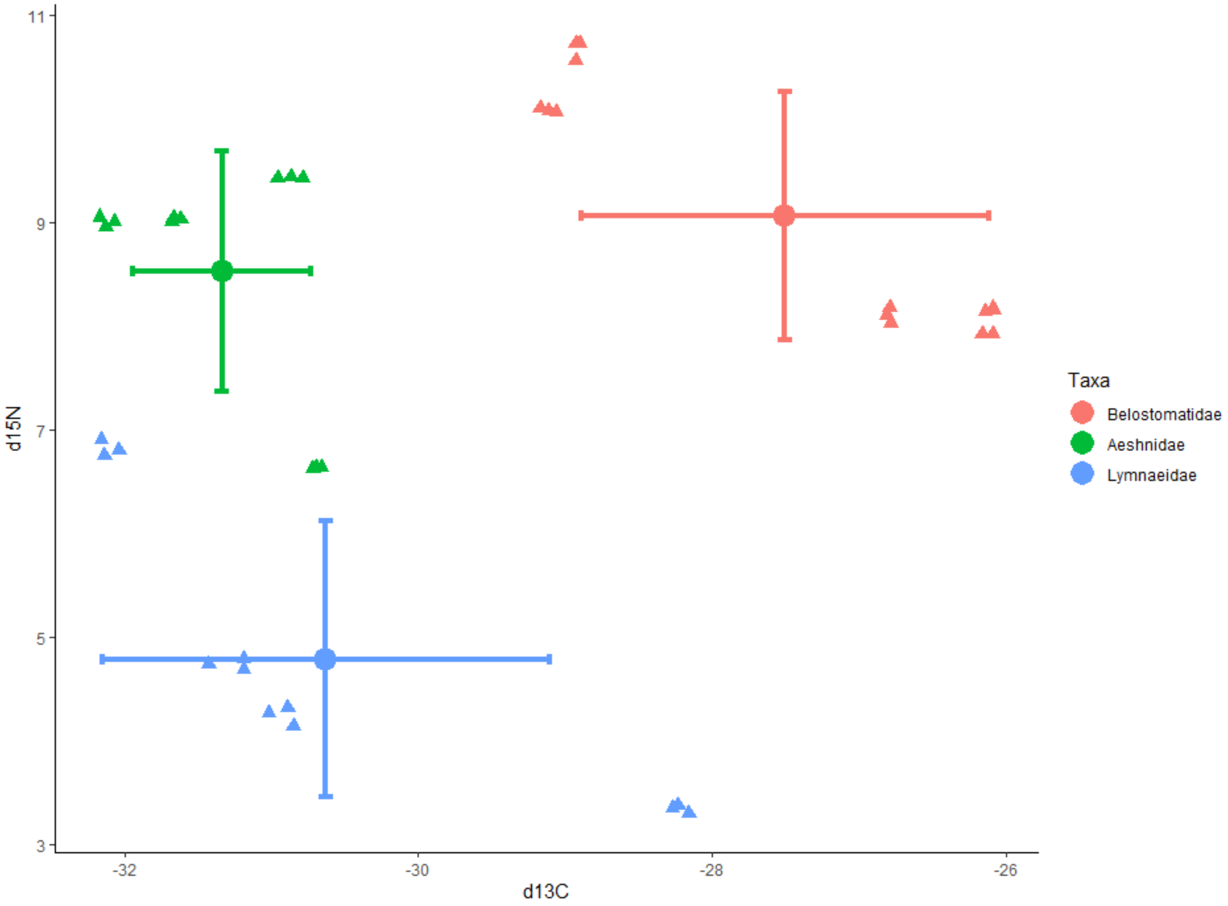

Figure S3: Stable isotope ($\delta^{15}N$, $\delta^{13}C$) signatures for selected taxa at Chalone Creek, South Wilderness of Pinnacles National Park, California (N: 39; 4 individuals for each taxa, with 3 analytical replicates each). $\delta^{13}C$ signatures for belostomatids diverge from *Aeschna* darner dragonflies (Odonata), the alternative top predator in fishless, intermittent sites. Furthermore, the observation that $\delta^{13}C$ values of Lymnaeidae snails matches $\delta^{13}C$ values of *Aeschna* dragonflies, but not of belosomatids, confirms that *Abedus*, at least seasonally, relied on terrestrially derived organic matter (less depleted $\delta^{13}C$ values) rather than on the 'green' or algal pathway (more depleted $\delta^{13}C$ values).

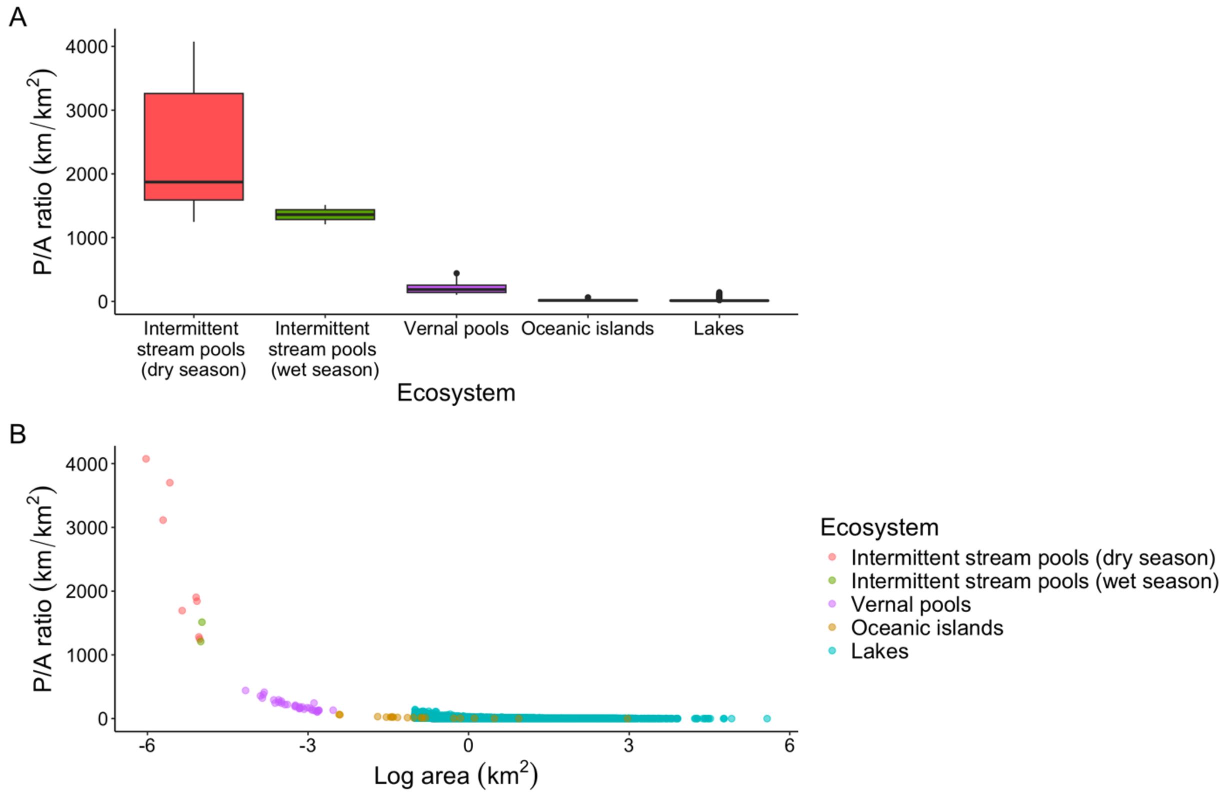

Figure S4: Perimeter to Area (P/A) ratios, and association between P/A ratios and areas, across a range of ecosystem types. (A) P/A ratios of 6 intermittent stream pools in our study area (Chalone Creek watershed, Pinnacles National Park, California), visited during the wet and dry seasons. We also show literature P/A values for oceanic islands (n=19; Polis et al. 1998), vernal pools (n=34; Brooks and Hayashi 2002), and a global data set on lakes (n=1,420,891; Messager et al. 2016). All P/A ratios are in km/km2, following Polis' et al. 1998 convention, for the sake of comparison. **(B)** Relationship between ecosystem surface areas (log km^2) and P/A ratios across ecosystem types, showing that intermittent stream pools are substantially smaller and have much higher P/A ratios (5-to-350 fold) than any other ecosystem type considered here, particularly during their dry phase.

Supplementary references:

Brooks, R. T., & Hayashi, M. (2002). Depth-area-volume and hydroperiod relationships of ephemeral (vernal) forest pools in southern New England. Wetlands, 22(2), 247-255.

Luce, R. D. (1977). The choice axiom after twenty years. Journal of Mathematical Psychology, 15(3), 215–233.

Hódar, J. A. (1996). The use of regression equations for estimation of arthropod biomass in ecological studies. *Acta (Ecologica, 1996, 17 (5), 421*, 433.

Benke, A. C., Huryn, A. D., Smock, L. A., & Wallace, J. B. (1999). Length-mass relationships for freshwater macroinvertebrates in North America with particular reference to the southeastern United States. *Journal of the North American Benthological Society*, *18*(3), 308-343.

Messager, M.L., Lehner, B., Grill, G., Nedeva, I., Schmitt, O. (2016): Estimating the volume and age of water stored in global lakes using a geo-statistical approach. Nature Communications: 13603. doi: 10.1038/ncomms13603. Data is available at www.hydrosheds.org.

Polis, G.A., Hurd, S.D., Jackson, C.T. and Sanchez-Piñero, F., 1998. Multifactor population limitation: variable spatial and temporal control of spiders on Gulf of California islands. *Ecology*, *79*(2), pp.490-502.

Sabo, J. L., Bastow, J. L., & Power, M. E. (2002). Length–mass relationships for adult aquatic and terrestrial invertebrates in a California watershed. *Journal of the North American Benthological Society*, *21*(2), 336-343.

Turner, H. L., Etten, J., Firth, D., & Kosmidis, I. (2020). Modelling Rankings in R: The PlackettLuce Package. Computational Statistics, 35, 1027–1057.
